# Cancer-associated Marantic Endocarditis and Direct Oral Anticoagulants: **Case Report and Systematic Review**

**DOI:** 10.2174/011573403X371101250729223222

**Published:** 2025-08-05

**Authors:** Glaudir Donato, Filipe Castor, João Vitor Andrade Fernandes, Emilio Carlos de Arruda Lacerda, Ariane Vieira Scarlatelli Macedo, Marcelo Melo

**Affiliations:** 1 Center of Medical Sciences, Federal University of Paraíba, João Pessoa, Brazil;; 2 Department of Oncology, Napoleão Laureano Hospital, João Pessoa, Brazil;; 3 Faculty of Medical Sciences of Santa Casa de São Paulo, São Paulo (SP), Brazil;; 4 Internal Medicine Department, Federal University of Paraíba, João Pessoa, Brazil

**Keywords:** Non-infective endocarditis, neoplasms, anticoagulants, transesophageal echocardiography, three-dimensional echocardiography, marantic endocarditis

## Abstract

**Introduction:**

Nonbacterial thrombotic endocarditis (NBTE) involves sterile vegetations on heart valves due to systemic hypercoagulability, often linked to malignancies. It is frequently underdiagnosed and presents significant clinical management challenges, often manifesting as thromboembolic events like ischemic stroke. We aim to report the first case of cancer-associated NBTE that showed a positive response to rivaroxaban as a maintenance anticoagulant, with 3D echocardiographic evidence of vegetation reduction, and to evaluate the performance of direct oral anticoagulants (DOACs) in treating cancer-associated NBTE through a systematic review of case reports.

**Methods:**

A case study and a systematic review of case reports on the use of DOACs in the setting of cancer-associated non-valvular atrial fibrillation were presented. Electronic databases were searched, and relevant studies were selected based on predefined eligibility criteria. Data were extracted and analyzed qualitatively, focusing on demographics, clinical presentation, anticoagulation and anticancer therapies, and outcomes (surgery, NBTE resolution, thromboembolic events, and mortality).

**Results and Discussion:**

In addition to our case, thirty-three studies were included in the systematic review. Most patients were already on DOAC therapy before the NBTE diagnosis. Lung and pancreatic cancers were the most common primary neoplasms. The aortic and mitral valves were most frequently affected. Anticoagulation therapy often shifted from DOACs to heparin upon NBTE diagnosis, with low-molecular-weight heparin showing better outcomes in vegetation resolution and thromboembolic event prevention.

**Discussion:**

This study reinforces the suggestion that heparins are more appropriate for treating cancer-associated NBTE. However, satisfactory DOAC outcomes warrant further research.

**Conclusion:**

Effective NBTE management with DOACs appears to be strongly associated with the successful control of the underlying neoplasm.

## 1 INTRODUCTION

Nonbacterial thrombotic endocarditis (NBTE), also known as Marantic endocarditis or non-infective endocarditis, was first described in 1888 by Ziegler, who initially termed it thromboendocarditis [1]. It involves the formation of sterile vegetations on previously healthy heart valves, resulting from a state of systemic hypercoagulability [2, 3]. This pathology is associated with underlying clinical conditions, notably advanced neoplasms, followed by autoimmune diseases such as antiphospholipid syndrome, systemic lupus erythematosus, and rheumatoid arthritis [4-7].

In addition to being frequently underdiagnosed and often only detected post-mortem, NBTE poses challenges for the clinical management of patients [8]. The entity manifests as thromboembolic complications, with ischemic stroke being the most common and typically the initial presentation. Other recurrent complications include renal and splenic infarcts [9]. Therefore, anticoagulation is one of the cornerstones of NBTE treatment. However, the literature lacks studies evaluating the efficacy of direct oral anticoagulants (DOACs) compared to other therapeutic modalities, such as vitamin K antagonists or heparin. Studies published to date have suggested better performance by the latter, whether unfractionated or low-molecular-weight heparin (LMWH) [2, 3, 10, 11].

This article aims to contribute to the ongoing discussion by reporting the first case of NBTE linked to metastatic lung adenocarcinoma with a good response to rivaroxaban as a maintenance anticoagulation, along with echocardiographic evidence of a reduction in valvular vegetations. In line with this, a systematic review of other cases using DOACs in the context of NBTE caused by cancer deepens the debate on the adequacy of this therapy for managing hypercoagulability scenarios.

### 1.1 Case Report

We report a case of an 82-year-old Black South American male patient without prior comorbidities. The patient presented with progressive left hip pain over three months, leading to an inability to walk. He denied weight loss, fever, or other joint or systemic symptoms. Upon seeking urgent medical attention for pain relief, a hip X-ray revealed a large tumor near the acetabulum. After pain management, the patient was referred for oncology evaluation, during which a low-dose chest computed tomography scan detected a pulmonary mass.

**Fig. (1):**
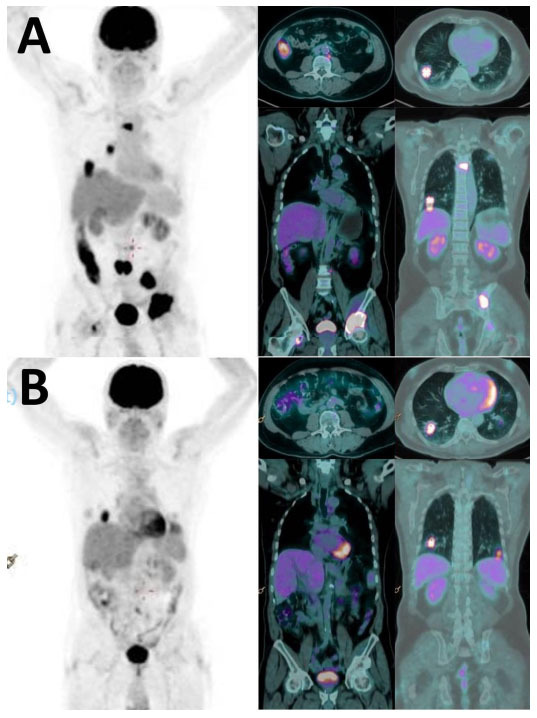
Baseline and follow-up PET-CT. (**A**) PET-CT/18F-FDG before immunotherapy showing the primary lung lesion and metastases, with the most prominent in the left hip (February 2019). (**B**) Follow-up PET-CT/18F-FDG showing mild lung lesion regression, fewer active metastatic sites, and an increment in myocardial 18F-FDG uptake (October 2019).

Further staging with positron emission tomography-computed tomography (PET-CT) scan and metabolic study with 18F-fluorodeoxyglucose (18F-FDG) identified secondary bone lesions and confirmed the lung lesion (Figs. **1A** and **1B**). A lung lesion biopsy was performed, and histopathology confirmed metastatic undifferentiated adenocarcinoma with PD-1 protein overexpression. Immunotherapy with pembrolizumab and denosumab was initiated.

**Fig. (2):**
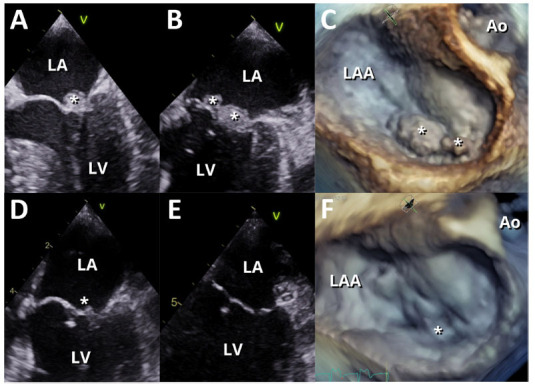
Baseline and follow-up 2D and 3D echocardiograms. Two-dimensional echocardiogram in the apical 4- (**A**) and 2-chamber (**B**) views, showing a vegetative lesion on the posterior cusps, P2 and P3. (**C**) Three-dimensional transesophageal echocardiogram (TEE) showing vegetative lesions on the posterior cusps in P2 and P3. Two-dimensional TEE after two weeks of subcutaneous low-molecular-weight heparin and one month of rivaroxaban treatment, demonstrating near-complete resolution of vegetations, in the apical 4- (**D**) and 2-chamber (**E**) views. (**F**) Three-dimensional TEE after two weeks of subcutaneous low-molecular-weight heparin and one month of rivaroxaban treatment, demonstrating near-complete resolution of vegetations. LA: left atrium; LAA: left atrial appendage; LV: left ventricle; Ao: aorta; (*): vegetations.

Although pain relief was achieved, two months later, the patient presented with sudden mental confusion and hemianopsia. Initial evaluations did not reveal metabolic, infectious, or electrolyte abnormalities. A cranial computed tomography confirmed an ischemic stroke in the left occipital region. Additional tests, including 24-hour Holter monitoring and magnetic resonance angiography of the cervical and cerebral vessels, were unremarkable. However, transesophageal echocardiogram (TEE) identified two amorphous, hyperechoic, sessile structures with slight mobility, measuring up to 2 cm, adherent to the posterior leaflet of the mitral valve (Figs. **2A**-**2C**).

Given the underlying oncological condition, the absence of clinical findings suggestive of infective endocarditis, and negative blood culture results, a diagnosis of NBTE was considered. Evaluation for other infectious causes of endocarditis with negative cultures also yielded normal results. Additionally, antibody testing ruled out associations with autoimmune conditions.

The patient received subcutaneous LMWH (80 mg twice daily) for two weeks. He remained stable with mild visual deficits and no further ischemic events. He was discharged on oral rivaroxaban at 15 mg once daily due to cost-effectiveness and ease of dosing. A month later, TEE showed near-complete resolution of vegetations, with a slight residual 5 mm hyperechoic, sessile structure adherent to the posterior cusp in P2 (Figs. **2D**-**2F**), and a reduction in valve thickness.

Clinical progress was favorable, with no new thromboembolic events. Pain improved, ambulation returned, and follow-up PET-CT confirmed the ongoing presence of the primary lung neoplasm, with a near-complete metabolic resolution of secondary lesions, indicating a partial but significant response to immunotherapy. Interestingly, there was also an increase in myocardial enhancement related to 18F-FDG uptake [12]. He passed away three years later due to oncologic progression. The anticoagulant therapy remained unchanged throughout this period (Table **1**).

## 2 METHODS

### 2.1 Ethical Considerations

The Ethics Committee of the Center of Medical Sciences at the Federal University of Paraíba approved the study (CAAE: 79053224.1.0000.8069, Approval Opinion No. 6.967.238), and waived the requirement for the informed consent form for the case report. Scientific committee approval was not required for the systematic review.

### 2.2 Systematic Review Methods

Following the Preferred Reporting Items for Systematic Reviews and Meta-Analyses (PRISMA) [13], a systematic review of case reports was performed to gather data on the use of DOACs in the context of cancer-related NBTE. This study was registered with the International Prospective Register of Systematic Reviews (PROSPERO) (CRD42024496124).

An electronic search was performed on December 21, 2023, using the Medical Literature Analysis and Retrieval System Online (MEDLINE), Excerpta Medica Database (EMBASE), and Web of Science. The search was updated on May 21, 2024. Medical Subject Headings (MeSH) and EmTree descriptors were used to develop the search strategy for each database, which is detailed in the Supplemental Methods.

### 2.3 Eligibility Criteria

Studies were included based on the PICOS criteria (population, intervention, comparison, outcomes, and study design) without language restrictions (Table **2**).

### 2.4 Study Selection

Two independent reviewers (G. D. and F. C.) screened titles and abstracts, with a third reviewer (M. M.) resolving conflicts. Full-text screening followed, again involving the third reviewer for conflict resolution. References were managed using the Covidence tool (Covidence Systematic Review Software, Veritas Health Innovation, Melbourne, Australia. Available at www.covidence.org.).

### 2.5 Data Collection and Evaluation

Data collection was performed by two independent reviewers (G. D. and F. C.) and conflicts were resolved by a third (M. M.). Extracted data were compiled into an online spreadsheet accessible to all authors.

Identification information (authors, year of publication, country of origin), clinical aspects (age, sex, past medical history, cancer diagnosis, clinical presentation on admission, imaging exams performed to identify vegetations, anticoagulation therapy, and, when present, ethnicity and anticancer therapy), and reported outcomes of interest (valve surgery, NBTE resolution, occurrence of thromboembolic events, and mortality— in-hospital, within 30 days, and within a year) were extracted from each included case.

### 2.6 Quality and Bias Assessment

Methodological quality was assessed using the JBI Critical Appraisal tools for case reports [14], scoring each study from 0 to 8. Studies were categorized as poor (0-2), fair (3-5), or good (6-8). Two reviewers (G. D. and F. C.) performed this assessment, with conflicts resolved by a third (M. M.).

### 2.7 Data Synthesis

A qualitative analysis of the demographics and clinical characteristics was performed. Continuous variables were reported as means and standard deviations or medians and interquartile ranges based on the normality assumption. Categorical variables were expressed as absolute and relative frequencies. Tables and timeline schemes were employed to compare the results and support conclusions.

## 3 RESULTS

### 3.1 Study Selection

**Fig. (3):**
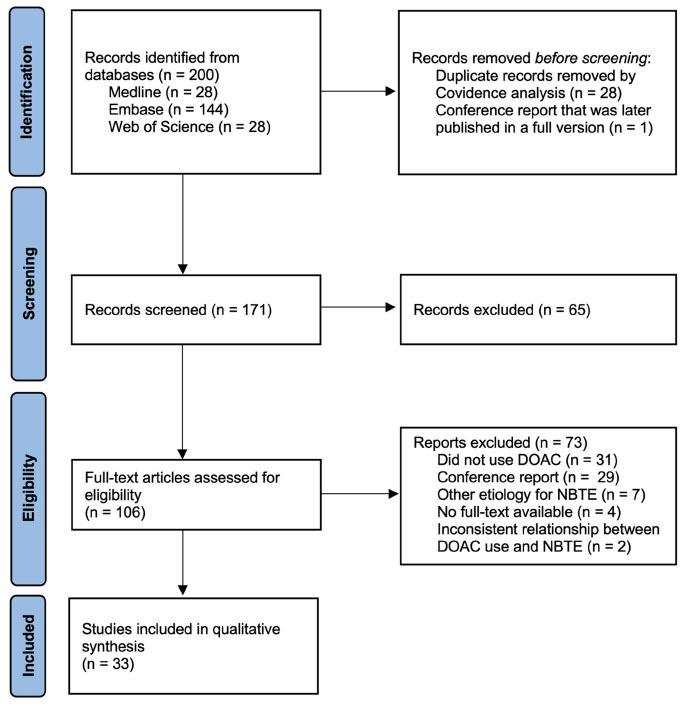
PRISMA flowchart. A flow diagram illustrating the studies screened, assessed for eligibility, and included in the review, along with the reasons for exclusions.

Our search strategy identified 200 studies, of which 106 were fully reviewed (Supplemental Table **1**). Seventy-three studies were excluded due to publication type, patient population, or intervention discrepancies, and three were excluded due to inaccessible full texts. This review includes 33 case reports on cancer-associated NBTE and DOACs (Fig. **3**) [15-47].

### 3.2 Quality and Bias Evaluation

The analysis found 29 studies (87.9%) rated as 'Good', meeting at least 6 of 8 quality criteria, while four studies (12.1%) were rated as 'Fair', meeting between 3 and 5 criteria. No study was rated as 'Poor'. The lowest success rate (57.6%) was for describing interventions and treatments performed, often lacking details on dosages, administration routes, and frequency of anticoagulant use. The second most common deficiency (30.3%) was in demographic details, such as ethnicity, medical history, comorbidities, and ongoing medication. Overall, studies successfully addressed the patient's disease history in an easily understandable chronology, describing the clinical condition of emphasis (NBTE) and diagnostic methods. Complete quality assessments for each study are available in Supplemental Figs. **1** and **2**.

### 3.3 Evidence Synthesis

Among the 33 patients, 19 were female (57.6%), and 14 were male (42.4%), aged 41 - 80 years, with a mean of 62.5 ± 9.86 years. Cases were from the USA (n=8), Japan (n=6), Belgium (n=4), Germany (n=3), France (n=2), Italy (n=2), Portugal (n=2), the UK (n=2), Austria (n=1), the Netherlands (n=1), Spain (n=1), and Switzerland (n=1). Baseline characteristics are available in Table **3**.

Lung cancer was the most frequent primary neoplasm (n=14, 42.4%), followed by pancreatic cancer (n=4, 12.1%) and endometrial cancer (n=3, 9.1%). One case had concurrent endometrial and ovarian cancers. Other primary diagnoses (bladder, cervical, cholangiocarcinoma, duodenal, esophageal, gastric, melanoma, and prostate) also appeared once each. In one-third of cases (n=11), no cancer treatment was described, and in three cases (9.1%), the cancer diagnosis was made post-mortem.

The most common clinical presentation during hospitalization when NBTE was diagnosed was stroke (n=18, 54.5%), followed by dyspnea (n=8, 24.2%) and renal infarction or ischemia (n=5, 15.2%). Table **4** summarizes these presentations and data on valve involvement, anticoagulant use before and after NBTE diagnosis, and the main outcomes.

In the process of identifying vegetations, the use of TTE was reported in 22 cases (66.7%), detecting vegetations in 20/22 (90.9% sensitivity). TEE was employed and revealed vegetative lesions in 30 cases (90.9%, with 100% sensitivity), mainly for detailed analysis after TTE findings. In two cases, the echocardiographic method used was unspecified. In one of these cases, the vegetations were only noticed during the autopsy.

The aortic and mitral valves were the most affected (20 cases each, 60.6%), while the tricuspid (n=2, 6.9%) and pulmonary valves (n=1, 3.4%) were the least affected. In eight cases, there was concurrent involvement of more than one valve, with six involving the aortic and mitral valves, one involving the mitral and tricuspid valves, and one involving all four cardiac valves.

The introduction of DOAC therapy occurred before NBTE was diagnosed in most cases (n=29, 87.9%). In this regard, previous episodes of deep vein thrombosis (n=16) and pulmonary embolism (n=14) were the main clinical indications, occurring concomitantly in most cases (n=9). Other indications included atrial fibrillation (n=7), ischemic stroke (n=5), and femoral artery stenosis (n=1). On the other hand, DOAC treatment began due to the diagnosis or suspicion of NBTE in four cases (12.1%), with one combining DOAC with clopidogrel (Table **5**).

Regarding the type of DOAC used, rivaroxaban was used in 15 patients (45.5%), apixaban in 12 (36.4%), and edoxaban in 6 (18.2%). Four patients switched DOACs even before the diagnosis of NBTE due to thromboembolic complications (n=3, switched from rivaroxaban to apixaban) or a suspected drug reaction (n=1, switched from edoxaban to rivaroxaban). Moreover, DOAC therapy was discontinued shortly before the NBTE diagnosis in two cases. After the NBTE diagnosis, 24 cases (72.7%) changed anticoagulation regimens (Graphical Abstract), with 79.2% (19/24) starting heparin therapy, usually LMWH (n=14).

Regarding the studied outcomes, surgical intervention was performed on the affected valve in five cases. Of these, one reported a new episode of deep venous thrombosis and thickening, and stenosis of the valve prosthesis after surgery, while another described a recurrence of vegetations affecting the valve prosthesis. A total of 12 non-surgical cases reported partial (n=2, 6.1%) or total resolution (n=10, 31.0%) of vegetations. Despite treatments, three cases (9.1%) had new thromboembolic episodes, and all-cause in-hospital mortality was 21.2%.

Table **5** summarizes all case reports included in this systematic review, including study and patient identification, clinical history, neoplasia diagnosis and treatment, the reason for DOAC initiation, clinical presentation on admission that prompted NBTE investigation, valve involvement, anticoagulation before and after diagnosis/identification of vegetations, and main outcomes. Supplementary Table **2** provides summary timelines for each case, highlighting the sequence of critical events and the therapies employed.

## 4 DISCUSSION

This article presents the first case report documenting the regression of cancer-associated NBTE using 3D echocardiography in a lung cancer patient, in which a DOAC was used as a maintenance anticoagulant. Moreover, it includes the first systematic review of case reports on the use of DOAC therapy in NBTE.

Consistent with over half of the NBTE cases in the literature, our patient was hospitalized due to an embolic ischemic stroke [9], as seen in 54.5% (n=18) of reviewed reports. These lesions often present with a multifocal pattern [48-50], as observed in 77.8% of these cases (n=14/18). However, our patient had a focal impairment, with clinical expression consistent with the affected brain region. In this context, identifying the etiology of the ischemic stroke became more challenging, requiring the exclusion of other more common causes like atherothrombosis [2].

As reaffirmed in this systematic review, TEE stands out in NBTE investigation, as it is highly sensitive for vegetation detection [51-54]. As an example of this relevance, in our case, TEE with 3D reconstruction identified valvular thrombotic foci, guiding clinical investigation toward an embolic origin. In this scenario, applying the Duke criteria to rule out infectious endocarditis and exploring other non-infective endocarditis etiologies is crucial [2, 6]. Other imaging modalities may further enhance this assessment, such as tissue Doppler imaging (TDI), which helps differentiate intracardiac masses from artifacts based on motion characteristics, expediting the detection of vegetations and thrombi [55, 56]. Moreover, pulsed wave TDI (PW-TDI) has demonstrated prognostic value, as higher peak antegrade velocities (≥10 cm/s) of intracardiac masses are associated with an increased risk of embolic events [57]. These tools contribute to a more precise evaluation of NBTE, improving both diagnosis and risk stratification [58].

Considering the highlighted clinical case, the patient's prior pulmonary malignancy diagnosis strongly supported the embolism suspicion [59, 60]. Generally, NBTE often complicates neoplasms, with adenocarcinomas of the lung and pancreas being the most frequent [61-63]. Our review findings confirm this epidemiology.

Furthermore, other aspects reported in the literature on NBTE that align with the reported case include the most common involvement of the mitral valve and the fact that this condition poses higher risks of thromboembolic events than in infective endocarditis [2, 52]. Regarding valvular involvement, our systematic review found an equal frequency of aortic and mitral valve involvement, unlike data in the literature [64, 65].

Treatment of NBTE typically involves three main strategies: anticoagulation [11], management of the underlying disease [4, 66], and valve surgery [67] if there is significant hemodynamic impact from valve involvement [68, 69]. Since the reported case did not meet this criterion, it did not require a surgical approach. Moreover, this review found that valve surgery is infrequently performed (15.2%) in similar cases due to its specific indications.

Regarding anticoagulation, in addition to the use of heparin in the hospital setting, a satisfactory response to DOACs can be inferred in our case. No new thromboembolic events occurred after the introduction of rivaroxaban, and echocardiography revealed vegetation reduction over a month.

This favorable response to rivaroxaban as maintenance anticoagulant therapy contrasts with most reports, as observed in this review [2, 10, 70].

Our review found that 87.9% (n=29) of patients were already on DOAC therapy even before the diagnosis of NBTE, indicating these agents might be insufficient for controlling hypercoagulability even at therapeutic dosages. Furthermore, thromboembolic events occurred in 58.6% of these cases (16/29) during DOAC therapy. This context justifies the high rate of anticoagulation therapy changes either before or after NBTE diagnosis (78.8%, 26/33), which suggests the good performance of heparin modalities. LMWH was the most commonly used and effective therapy, with partial resolution of vegetations in two reviewed cases and complete resolution in six of ten cases, all confirmed by echocardiography.

Within this discussion, a notable case by Schneider-Reigbert, M. (2022) [41] demonstrated NBTE recurrence with echocardiographic evidence on two occasions upon switching from LMWH to apixaban as maintenance anticoagulant therapy. Similarly, Panicucci *et al*. (2021) [38] reported recurrent strokes when switching from heparin to a DOAC. Similar failures were also observed with warfarin [28, 36].

It is argued that, given the physiopathological complexity of the paraneoplastic embolic scenario, the pleiotropic effects exerted by heparins, which irreversibly target factor Xa and thrombin, provide better efficacy compared to other therapies [71]. However, evidence indicates the non-inferiority of DOACs compared to LMWH in treating paraneoplastic venous thromboembolism [72].

Among the reviewed studies, only five described outcomes suggestively favorable to the use of DOACs. In Ahmed *et al*. (2018) [15], apixaban was introduced upon the diagnosis of NBTE associated with an underlying lymphoproliferative disorder, with an 18-month follow-up reporting stabilization of previous renal dysfunction. However, this follow-up description does not explore potential adverse events related to DOAC anticoagulation. On the other hand, Kaufmann *et al*. (2021) [26] observed control of the hypercoagulable state with rivaroxaban and clopidogrel after uncontrolled embolic episodes with LMWH. The nuance of this case management is the addition of an antiplatelet agent.

The effectiveness of oncological therapy is particularly noteworthy in the other three cases with satisfactory outcomes using DOACs [20, 27, 46]. This aspect was similarly observed in our reported case, as evidenced by new findings on PET-CT and the recovery of the patient's walking ability. We believe this responsiveness was crucial for the patient's prognosis in NBTE follow-up. Supporting this, among the reviewed cases with echocardiographic evidence of partial (n=2) or total (n=10) resolution of vegetations, 75% (9/12) were receiving anticancer therapy. Furthermore, a systematic review and meta-analysis of 416 NBTE cases indicated cancer as an etiology associated with a higher risk of morbidity and mortality compared to other etiologies [64].

Overall, there are few descriptions of success in oncological treatment, as most neoplasms are present in advanced stages [2, 4], with many cases discovered post-mortem, as observed in this review. This context underscores the need to explore the topic further to complement the discussion surrounding anticoagulants.

## CONCLUSION

To our knowledge, this article provides the first report of NBTE associated with lung cancer with a positive response to rivaroxaban as a maintenance anticoagulant therapy, documented by three-dimensional echocardiography. Additionally, this is the first systematic review investigating DOAC outcomes in cancer-associated NBTE. The results of this study reinforce the suggestion that heparins are more suitable for treating the paraneoplastic hypercoagulable state. Nevertheless, the occasional satisfactory outcomes with DOACs warrant further research to explore safe alternatives to long-term heparinization. Our theory is that, beyond anticoagulation, a significant part of therapeutic effectiveness is linked to successful control of the underlying neoplasm, another pillar of NBTE treatment. This underscores the need for a multidisciplinary approach, where oncological management plays a crucial role in preventing thrombotic recurrence and improving overall prognosis. Future studies should focus on defining specific scenarios where DOACs may be viable, considering tumor type, disease burden, and individual thrombotic risk factors.

## LIMITATIONS

This study has limitations due to its case report basis, preventing the establishment of causal relationships between clinical aspects and outcomes. Additionally, as outlined in the quality assessment and risk of bias evaluation, as well as in the summary tables of results, there are gaps in clinical descriptions, such as demographic characteristics, intervention details (*e.g.*, administration routes, dosage frequencies, side effects), and prognosis. These limitations, combined with a small sample size, hinder quantitative data synthesis. The sample limitation extends to the exclusion of unpublished cases or those presented only at conferences.

## AUTHORS’ CONTRIBUTIONS

The authors confirm their contribution to the paper: study conception and design: GD, FC, JVAF, ECAL, AVSM, MM. Data collection: GD, FC, JVAF, ECAL, MM; analysis and interpretation of results: GD, FC, JVAF, MM; draft manuscript: GD, FM, JVAF, MM. All authors reviewed the results and approved the final version of the manuscript.

## Figures and Tables

**Table 1:** Timeline of the main events of the case report.

**Relative Point in Time**	**Events**
2 months before admission	Diagnosis of metastatic undifferentiated lung adenocarcinoma. Initiation of immunotherapy using pembrolizumab and denosumab.
At admission	Sudden right homonymous hemianopsia. Cranial computed tomography confirmed an ischemic stroke in the left occipital region.
1^st^ week after admission	24-hour Holter and magnetic resonance angiography of cervical and cerebral vessels did not show any abnormalities. TEE identified vegetation on the posterior leaflet of the mitral valve.
2^nd^ week after admission	The hypothesis of NBTE was raised due to the absence of clinical findings of infective endocarditis and negative blood cultures. Investigation into the causes of negative-culture endocarditis ruled out other infectious and autoimmune causes. Initiation of low molecular weight heparin anticoagulation at a dose of 80 mg subcutaneously twice daily.
1 month after admission	Hospital discharge with rivaroxaban (15 mg orally once daily).
2 months after admission	Follow-up TEE showed near-complete resolution of vegetations.
6 months after admission	Follow-up PET-CT indicated a partial and significant response to immunotherapy.
3 years later	Death due to complications of the oncological condition.

**Abbreviations:** PET-CT: positron emission tomography and computed tomography; NBTE: nonbacterial thrombotic endocarditis; TEE: transesophageal echocardiography.

**Table 2:** Eligibility criteria according to PICOS.

**Parameter**	**Inclusion Criteria**	**Exclusion Criteria**
Population	Patients with cancer-associated NBTE	Studies involving animals, patients with other etiologies for NBTE
Intervention	Use of DOAC in the context of NBTE, before or after its diagnosis	Use of DOAC for other instances, inconsistent relationship between DOAC use and NBTE^a^, articles that did not use DOAC
Comparison	Not applicable	-
Outcomes	Not applicable	-
Study design	Case reports, case series	Conference reports, case-control, cohort, and cross-sectional studies

DOAC, direct oral anticoagulant; NBTE, nonbacterial thrombotic endocarditis.

^a^Case reports were excluded if the DOAC was discontinued long before the diagnosis of NBTE without a defined reason or due to bleeding events, for instance, which contrasted with the hypercoagulable scenario.

**Table 3:** Baseline characteristics of the review sample.

**Variable**	**-**	**Overall**
Sample	-	33
Age (Mean ± SD)	-	62.5 ± 9.86
Sex (%)	Female Male	19 (57.6%) 14 (42.4%)
Country (%)	USA Japan Belgium Germany France, Italy, Portugal, UK Austria, Netherlands, Spain, and Switzerland	8 (24.2%) 6 (18.2%) 4 (12.1%) 3 (9.1%) 2 each (6.1% each) 1 each (3.0% each)
Main past history clinical presentations (%)^a^	Deep vein thrombosis, Pulmonary embolism Smoking Cerebrovascular disease Atrial fibrillation, Hypertension Dyslipidemia, Thyroidopathy COPD, Renal infarction, Splenic infarction	16 each (48.5% each) 8 (24.2%) 7 (21.2%) 6 each (18.2% each) 4 each (12.1% each) 2 each (6.1% each)
Primary sites of neoplasia (%)	Lung cancer Pancreatic cancer Endometrial cancer^b^ Hematological cancer, Ovarian^b^ Others (bladder, cervical, cholangiocarcinoma, duodenal, esophageal, gastric, melanoma, prostate)	14 (42.4%) 4 (12.1%) 3 (9.1%) 2 each (6.1% each) 1 each (3.0% each)
Anticancer therapy Onset (%) Management (%)^c^	Overall Before the NBTE diagnosis After NBTE diagnosis Chemotherapy Immunotherapy Surgical intervention Radiotherapy Hormone therapy	23 (69.7%) 12/23 (52.2%) 11/23 (47.8%) 17/23 (73.4%) 9/23 (39.1%) 5/23 (21.7%) 1/23 (4.3%) 1/23 (4.3%)

**Abbreviations:** COPD: chronic obstructive pulmonary disease; NBTE: nonbacterial thrombotic endocarditis; SD: standard deviation.

**Note:** ^a^Past medical history included any known acute event or chronic condition before the hospitalization in which NBTE was diagnosed. This aspect was not mentioned in all cases. In some cases, there was a concomitant occurrence of more than one previous clinical condition, so the sum of the frequencies is greater than 1.

^b^In one case, there were concomitant primary sites of endometrial and ovarian neoplasia.

^c^In ten cases, more than one anticancer therapy was used simultaneously or at different moments, so the sum of the frequencies was greater than 1.

**Table 4:** Clinical presentations on admission, affected valves, anticoagulants before and after NBTE diagnosis, and outcomes were studied.

**Variable**			**Overall**
**Sample**			**33**
Main clinical presentations on admission (%)^a^	Stroke Renal infarction/ischemia Splenic infarction/ischemia Limb ischemia DIC Nonspecific Dyspnea Fever Peripheral edema		18 (54.5%) 5 (15.2%) 4 (12.1%) 3 (9.1%) 2 (6.1%) 8 (24.2%) 4 (12.1%) 3 (9.1%)
Affected valves (%)^b^	Aortic Mitral Tricuspid Pulmonary		20 (60.6%) 20 (60.6%) 2 (6.1%) 1 (3.0%)
Anticoagulation and/or antiaggregation (%) Before NBTE diagnosis^c^	DOAC Heparin Aspirin None	Overall Rivaroxaban Apixaban Edoxaban Overall LMWH UFH Unspecified Overall Overall	**29 (87.9%)** 15/29 12/29 6/29 **9 (27.3%)** 5/9 3/9 2/9 **1 (3.0%)** **2 (6.1%)**
After NBTE diagnosis^d^	Heparin DOAC VKA Aspirin Clopidogrel Not reported	Overall LMWH Unspecified UFH Overall Apixaban Rivaroxaban Edoxaban Overall Overall Overall Overall	**24 (72.7%)** 18/24 6/24 4/24 **6 (18.2%)** 4/6 1/6 1/6 **4 (12.1%)** **2 (6.1%)** **1 (3.0%)** **1 (3.0%)**
Main outcomes (%)	Valve surgery Vegetation resolution without surgery^e^ New embolic event Mortality	Overall Replacement Vegectomy Complete Partial Not resolved Not reported In-hospital 30-day 1-year Unspecified Not reported	**5 (15.2%)** 3/5 2/5 10 (30.3%) 2 (6.1%) 5 (15.2%) 11 (33.3%) 3 (9.1%) 7 (21.2%) 8 (24.2%) 11 (33.3%) 1 (3.0%) 21 (63.6%)

**Abbreviations:** DIC: disseminated intravascular coagulation; DOAC: direct oral anticoagulant; UFH: unfractionated heparin; LMWH: low-molecular-weight heparin; NBTE: nonbacterial thrombotic endocarditis; VKA, vitamin K antagonist.

**Note:** ^a^As there was a concomitant occurrence of more than one clinical presentation in some cases, the sum of the frequencies is greater than 1.

^b^Multivalvular involvement occurred in eight cases: mitral and aortic (n=6), mitral and tricuspid (n=1), quadrivalvular (n=1). Two other cases affected an aortic bioprosthesis.

^c^In ten cases, more than one anticoagulant was used simultaneously or at different moments, so the sum of the frequencies was greater than 1.

^d^In twelve cases, more than one anticoagulant was used simultaneously or at different moments, so the sum of the frequencies was greater than 1.

^e^Complete or partial resolution attested by echocardiographic evidence.

**Table 5:** Summary of evidence drawn from studied case reports.

**Author (Year)**	**Patient Identification**	**Past History**	**Primary Neoplastic Site**	**Cancer Therapy**	**Reason for Introducing DOAC**	**Clinical Presentation on Admission and During Hospitalization**	**Affected Valve(s)**	**Anticoagulation**		**Main Outcomes**
								**Before the NBTE Diagnosis**	**After the NBTE Diagnosis**	
Ahmed, S. *et al*. (2018) [15]	Male, 71 y/o, USA	Acute kidney injury	T-cell large granular lymphocytic leukemia	Methotrexate → Cyclophosphamide	NBTE and AF diagnosis	Unexplained fatigue, dyspnea, and atrial fibrillation	Mitral	None	Apixaban (therapeutic dosage)	Renal function stabilized during an 18-month follow-up. No thromboembolic event was reported during this period.
Akiki, E. *et al*. (2023) [16]	Female, 70 y/o, USA	AF, malignant pericardial effusion	Metastatic lung adenocarcinoma	Pembrolizumab and pemetrexed	Previous AF	Multiple embolic strokes	Mitral	Apixaban (5 mg bid)	LMWH (70 mg bid)	NBTE resolution 4 weeks after starting LMWH.
Assaf, S. *et al*. (2023) [17]	Male, 76 y/o, USA	AF, HFpEF, CAD	Metastatic pancreatic cancer	Not reported	Previous AF	Hyponatremia and pneumonia	Mitral and tricuspid	Apixaban	LMWH	Death a few days after NBTE diagnosis.
Casal-Lorenzo, J. *et al*. (2021) [18]	Female, 80 y/o, Spain	HTN, dyslipidemia, chronic venous insufficiency, DVT	Endometrial cancer	Not reported	Previous DVT	Confusion syndrome, left MCA ischemic stroke, left kidney and spleen infarcts	Mitral and aortic	Apixaban (5 mg bid)	LMWH (weight-based dose)	NBTE resolution 18 days after starting LMWH.
Collienne, M. *et al*. (2020) [19]	Male, 51 y/o, Germany	DVT	Metastatic pancreatic cancer	Folforinox (leucovorin, 5-fluorouracil, irinotecan, oxaliplatin) chemotherapy *via* Port-A-Cath system	Previous DVT	Multiple embolic strokes, superficial thrombophlebitis, and splenic infarction	Aortic	Rivaroxaban (15 mg bid → 20 mg sid)	LMWH	No information reported on surgical interventions, resolution of NBTE, or death.
Douin, C. *et al*. (2020) [20]	Male, 75 y/o, Belgium	AF	Metastatic melanoma	Pembrolizumab	Previous AF	Progressive dyspnea	Aortic biological prosthesis	Edoxaban (60 mg sid)	LMWH	Aortic stenosis improvement after starting LMWH.
Duarte, F. *et al*. (2024) [21]	Male, 57 y/o, Portugal	Smoking	Diffuse large B-cell lymphoma	R-CHOP (rituximab plus cyclophosphamide, doxorubicin, vincristine, and prednisone)	Suspicion of NBTE	2-month history of unintentional weight loss, asthenia, painful and bilateral digital edema, and episodes of color change (from pallor to cyanosis)	Mitral	None	Apixaban + Low-dose aspirin	Persistence of mitral vegetation without valvular dysfunction or embolic events under apixaban. Favorable clinical evolution and stability during follow-up after starting chemotherapy.
Fujimoto, D. *et al*. (2018) [22]	Male, 65 y/o, Japan	PE, DVT	Bladder cancer	Cystectomy and ureterostomy	Previous DVT and PE	Left parietal lobe infarction and left angular gyrus subacute infarction	Mitral	Rivaroxaban (30 mg sid for 3 weeks)	Not reported	Vegectomy post-NBTE diagnosis.
Gabelmann, V. *et al*. (2021) [23]	Female, 52 y/o, Germany	Ischemic stroke, PE	Advanced clear cell cervical carcinoma (FIGO IV4A)	Lymphadenectomy + platinum-containing radiochemotherapy and navelbine	Previous PE	Tachycardia and hypotension	All of them	Edoxaban	UFH	Death 2 days after starting UFH.
Gundersen, H. *et al*. (2016) [24]	Afro-Caribbean male, 59 y/o, UK	Smoking (40 pack-year), DVT, PE	Squamous cell lung adenocarcinoma	Not reported	Previous DVT and PE	Multiple ischemic strokes, DIC, malignant MCA syndrome	Aortic	Rivaroxaban → heparin	Not applicable	Death 5 days after admission. Postmortem NBTE diagnosis. The autopsy also revealed squamous cell adenocarcinoma of the lung, infarcts in the brain, spleen, and kidneys.
Hedin, T. *et al*. (2023) [25]	Caucasian male, 54 y/o, USA	Obesity, AF, HFpEF	Poorly differentiated metastatic esophageal adenocarcinoma	Not reported	Previous AF	Atrial flutter, left pleuritic pain radiating to the lower back, 2 foci of subacute cerebral embolism	Mitral	Rivaroxaban	LMWH (full dose)	Ten-day hospital course and death 12 days after discharge.
Kaufmann, C.C. *et al*. (2021) [26]	Female, 44 y/o, Austria	Hypothyroidism, multiple strokes, PE	Metastatic lung adenocarcinoma	Osimertinib	NBTE diagnosis	Multiple ischemic strokes, renal and splenic infarctions	Aortic	LMWH (therapeutic dose bid)	Rivaroxaban (15 mg sid) + Clopidogrel (75 mg sid)	No thromboembolic events during a 10-month follow-up.
Kawakami, T. *et al*. (2024) [27]	Male, 56 y/o, Japan	Asthma, Smoking (27 pack-year)	Metastatic lung adenocarcinoma	Pembrolizumab → Pemetrexed and bevacizumab	NBTE treatment	Back pain (arterial thromboembolisms in the right kidney and spleen)	Aortic	UFH (20,000 IU/day IV → 18,000 IU/day SC)	UFH (20,000 IU/day IV → 18,000 IU/day SC) → Apixaban (5 mg bid)	Evidence of NBTE resolution 6 months after starting apixaban. No new thromboembolism during a 52-month follow-up.
Kuipers, R.S. *et al*. (2021) [28]	Female, 49 y/o, Netherlands	Migraine, recurrent DVT, PE and uterine myomas	Low-grade ovarian adenocarcinoma	Platinum-based chemotherapy, oophorectomy, salpingectomy, hysterectomy, omentectomy, and stripping of the bladder peritoneum	Previous PE and DVT	Loss of consciousness and VF	Aortic	Rivaroxaban (20 mg sid → 10 mg sid → 20 mg sid)	LMWH (175 IU/kg) → VKA → LMWH	DVT recurrence after starting VKA. NBTE resolution 10 months after restarting LMWH.
Lepour, M. *et al*. (2022) [29]	Male, 60 y/o, Belgium	Multiple ischemic strokes and AF	Poorly differentiated metastatic pancreatic adenocarcinoma	Gemcitabine	Previous ischemic stroke and AF	Progressive dyspnea, lower limb edema, and DIC	Aortic and mitral	Apixaban (5 mg bid)	LMWH	NBTE resolution 8 weeks after starting LMWH. No thromboembolic events during a 3-month follow-up. Death due to cancer progression 5 months after initial diagnosis, without signs of relapse of cardiac failure.
Lobo Ferreira, T. *et al*. (2017) [30]	Female, 67 y/o, Portugal	HTN, dyslipidemia, PE with lung infarction, DVT	Papillary adenocarcinoma of the lung	Not reported	Previous PE and DVT	Fever, sputum-productive cough, confusion, respiratory alkalosis with alkalemia, pleural effusion, pleural nodules, ureteropelvic junction stenosis causing left hydronephrosis	Aortic	LMWH → Rivaroxaban → LMWH (therapeutic dosage)	LMWH (prophylactic dosage)	Death 18 days after admission. Postmortem cancer diagnosis.
Mantovani, F. *et al*. (2016) [31]	Female, 65 y/o, Italy	PE and DVT	Pancreatic cancer stage T2M1N1	Not reported	Previous PE and DVT	Progressive dyspnea	Aortic and mitral	Rivaroxaban (20 mg sid)	UFH → LMWH (8,000 U SC bid)	Death 3 months after discharge with LMWH, 4 months after NBTE diagnosis.
McBriar, D. *et al*. (2020) [32]	Female, 72 y/o, UK	Mitral regurgitation, AF, COPD, hyperthyroidism	Squamous cell lung carcinoma	Not reported	Previous AF	Progressive dyspnea and peripheral edema	Mitral	Apixaban	Warfarin	No thromboembolic events for 2 months before discharge.
McCullough, J. *et al*. (2022) [33]	Female, 66 y/o, USA	Smoking (30 pack-year), HTN, dyslipidemia, right foot blue toe syndrome	Metastatic lung adenocarcinoma	Pembrolizumab	Common femoral and superficial femoral artery stenosis	Multiple strokes (aphasia) and dyspnea	Mitral	Apixaban + aspirin	IV heparin → LMWH (therapeutic dose)	No thromboembolic events reported after discharge with LMWH for a 15-month follow-up.
Murata, A. *et al*. (2022) [34]	Female, 48 y/o, Japan	No particular medical history	Lung adenocarcinoma	Unspecified chemotherapy	Ischemic stroke and PE	Ischemic stroke and PE	Aortic	Edoxaban (60 mg)	UFH	NBTE resolution 33 days after admission.
Nadkarni, N. *et al*. (2019) [35]	Female, 41 y/o, USA	Steroid-dependent rheumatoid arthritis, cerebellar and cerebral strokes, DVT	Metastatic cholangiocarcinoma	Unspecified palliative chemotherapy	Previous DVT	New systolic murmur	Aortic and prosthetic aortic	Apixaban	LMWH	No thromboembolic events reported after starting LMWH.
Nishimura, T. *et al*. (2020) [36]	Female, 66 y/o, Japan	Smoking (34 pack-year), DVT	Lung adenocarcinoma (cT2aN3M0, IIIB)	4 chemotherapy cycles with Pemetrexed and carboplatin → 9 cycles with Pemetrexed → Atezolizumab → Docetaxel	Previous DVT	Right brachial artery embolism (right arm monoparesis), ascending colon infarction (fecal blood), right kidney infarction, multiple strokes	Mitral	Edoxaban (30 mg sid), discontinued due to colon bleeding	Unspecified heparin (15,000 IU/day → 20,000 IU/day) + warfarin → warfarin→ Unspecified heparin	Mitral valve replacement after diagnosis. Vegetation recurrence after discontinuation of heparin. Discharge 36 days after restarting heparin.
Orfanelli, T. *et al*. (2016) [37]	Female, 63 y/o, USA	PE	High-grade papillary serous ovarian adenocarcinoma, stage IIC, and stage IA endometrial adenocarcinoma	Exploratory laparotomy and optimal cytoreduction of left ovarian mass; paclitaxel and carboplatin	Previous PE	Postmenopausal bleeding	Aortic and mitral	Rivaroxaban	Unspecified heparin → LMWH (full dose)	NBTE resolution after starting LMWH.
Panicucci, E. *et al*. (2021) [38]	Female, 64 y/o, France	Active smoking, thyroidopathy, DVT, PE	Metastatic lung adenocarcinoma	Not reported	Previous PE, DVT	Multiple strokes (sudden left hemiparesis and dysarthria)	Aortic	Rivaroxaban (15 mg bid) → UFH (therapeutic doses) → apixaban (5 mg bid) → LMWH (0.6 mL bid)	LMWH (0.6 mL bid)	No thromboembolic events after starting LMWH. Death occurred a few days after the cancer diagnosis, before starting chemotherapy.
Perrone, F. *et al*. (2020) [39]	Female, 63 y/o, Italy	Smoking (20 pack-year), renal infarction, splenic infarction, PFO, DVT, PE, and ischemic stroke	Lung adenocarcinoma stage IIIB	Not reported	Previous DVT	Diplopia, gait impairment (evolution of ischemic stroke), substernal chest pain, and dyspnea (acute pulmonary edema, STEMI)	Aortic and mitral	Rivaroxaban (10 mg sid) → LMWH (6,000 IU bid)	LMWH (6,000 IU bid) + aspirin	Ischemic strokes, bowel infarction, and peripheral embolization after LMWH. Death with multi-organ failure 70 days after cancer diagnosis.
Polo, J. *et al*. (2021) [40]	Male, 75 y/o, France	Smoking (not reported tobacco load), COPD, DVT, and PE	Advanced lung adenocarcinoma (T3M3N1a)	Paclitaxel and carboplatin	Previous PE and DVT	Lower left limb acute ischemia due to popliteal artery occlusion	Mitral	Rivaroxaban → LMWH (1 mg/kg bid)	LMWH (1 mg/kg bid)	Significant regression of vegetation 15 days after starting LMWH. Death 4 months after NBTE diagnosis due to cancer treatment failure.
Schneider-Reigbert, M. (2022) [41]	Female, 49 y/o, Germany	PE	Endometrial cancer	Not reported	Previous PE	Not reported	Aortic	Apixaban	LMWH → Apixaban → LMWH	NBTE resolution 5 weeks after starting LMWH. NBTE recurrence 5 weeks after restarting Apixaban. New NBTE resolution after restarting LMWH in a 4-month follow-up.
Shoji, M.K. *et al*. (2019) [42]	Female, 63 y/o, USA	Sigmoid colon cancer, HTN, dyslipidemia, DVT, PE, multiple ischemic strokes	Non-mucinous metastatic biliary adenocarcinoma	Unspecified chemotherapy	Previous PE, DVT	DRESS (fever, rash) and ischemic stroke (left hemiparesis, lethargy, confusion, dysarthria)	Aortic and mitral	Rivaroxaban → apixaban	Unspecified heparin → LMWH	Apparent stability of the NBTE after starting heparin.
Soga, Y. *et al*. (2018) [43]	Male, 69 y/o, Japan	Stroke, DVT	Gastric adenocarcinoma	Billroth I distal gastrectomy and chemotherapy	Previous Stroke and DVT	Stroke (right arm asthenia and dysarthria)	Mitral	Apixaban → Unspecified heparin	Unspecified heparin	Vegectomy on day 5 after referral. No thromboembolic events in an 18-month follow-up after discharge.
Tamura, Y. (2021) [44]	Female, 62 y/o, Japan	Not reported	Metastatic lung adenocarcinoma	Not reported	Presumed paradoxical embolism	Fever, multiple strokes (mild dysarthria), and right kidney infarction	Aortic	Edoxaban (30 mg) → rivaroxaban (15 mg)	Not applicable	Death on day 31 of hospitalization, one day after hemorrhagic stroke. Postmortem NBTE diagnosis.
Tisch, C. *et al*. (2024) [45]	Caucasian female, 70 y/o, Switzerland	HTN, Hashimoto thyroiditis, vitamin B12 deficiency, PE, renal and splenic arterial infarctions, DVT	Metastatic duodenal adenocarcinoma	Fluorouracil, folinic acid, irinotecan, and aflibercept	Previous DVT	Multiple strokes (memory deficits, left homonymous hemianopia, and personality changes)	Mitral	Rivaroxaban → Apixaban → UFH → Apixaban	Not applicable	Progression of mitral regurgitation and death on day 19 of hospitalization due to severe heart failure. Postmortem NBTE diagnosis.
Van Herck, J. (2021) [46]	Caucasian Male, 66 y/o, Belgium	PE, DVT	Metastatic prostate adenocarcinoma	Androgen deprivation therapy, denosumab	Previous PE, DVT	Multiple ischemic strokes (subacute vertigo, diplopia, dysbasia, and dysmetria)	Aortic	Edoxaban (60 mg sid), discontinued at home a week before the stroke episode	Edoxaban	NBTE resolution 5 months after restarting Edoxaban.
Zaher, W. (2023) [47]	Female, 74 y/o, Belgium	HTN, persistent fever, lymphadenopathy, PE	Metastatic lung carcinoma	Pembrolizumab → Chemotherapy	Previous PE	Fever, persistent dyspnea, amaurosis, diplopia (ischemic stroke)	Mitral	Rivaroxaban	IV heparin (50,000 U sid for 50 kg) → VKA	NBTE resolution 2 weeks after admission.

**Abbreviations:** AF, atrial fibrillation; bid: twice a day; COPD, chronic obstructive pulmonary disease; DOAC, direct oral anticoagulants; DVT, deep venous thrombosis; HFpEF, heart failure with preserved ejection fraction; HTN, hypertension; IHD, ischemic heart disease; IV, intravenous; LMWH, low-molecular-weight heparin; MCA, middle cerebral artery; NBTE, nonbacterial thrombotic endocarditis; PE, pulmonary embolism; PFO, patent foramen ovale; SC, subcutaneous; sid: once a day; STEMI, ST-elevation myocardial infarction; UNF, unfractionated heparin; VKA, vitamin K antagonist; VF, ventricular fibrillation; y/o, years old; →: therapy substitution.

## Data Availability

The data and supportive information are available within the article.

## LIST OF ABBREVIATIONS

18F-FDG: 18F-Fluorodeoxyglucose
DOAC: Direct-acting Oral Anticoagulant
LMWH: Low-molecular-weight Heparin
NBTE: Nonbacterial Thrombotic Endocarditis
PET-CT: Positron Emission Tomography and Computed Tomography
TEE: Transesophageal Echocardiography
TTE: Transthoracic Echocardiography

## STANDARDS OF REPORTING

PRISMA and CARE guidelines were followed.

## SUPPLEMENTARY MATERIAL

PRISMA and CARE checklists are available as supplementary material on the publisher’s website along with the published article.
